# The burst gap is a peripheral temporal code for pitch perception that is shared across audition and touch

**DOI:** 10.1038/s41598-022-15269-5

**Published:** 2022-06-30

**Authors:** Deepak Sharma, Kevin K. W. Ng, Ingvars Birznieks, Richard M. Vickery

**Affiliations:** 1 School of Medical Sciences, UNSW Sydney, Kensington, NSW 2052 Australia; 2 Neuroscience Research Australia, Randwick, NSW 2031 Australia; 3 Department of Biomedical and Clinical Sciences, Centre for Social and Affective Neuroscience, Linköping University, 581 83 Linköping, Sweden

**Keywords:** Neuroscience, Physiology

## Abstract

When tactile afferents were manipulated to fire in periodic bursts of spikes, we discovered that the perceived pitch corresponded to the inter-burst interval (burst gap) in a spike train, rather than the spike rate or burst periodicity as previously thought. Given that tactile frequency mechanisms have many analogies to audition, and indications that temporal frequency channels are linked across the two modalities, we investigated whether there is burst gap temporal encoding in the auditory system. To link this putative neural code to perception, human subjects (n = 13, 6 females) assessed pitch elicited by trains of temporally-structured acoustic pulses in psychophysical experiments. Each pulse was designed to excite a fixed population of cochlear neurons, precluding place of excitation cues, and to elicit desired temporal spike trains in activated afferents. We tested periodicities up to 150 Hz using a variety of burst patterns and found striking deviations from periodicity-predicted pitch. Like the tactile system, the duration of the silent gap between successive bursts of neural activity best predicted perceived pitch, emphasising the role of peripheral temporal coding in shaping pitch. This suggests that temporal patterning of stimulus pulses in cochlear implant users might improve pitch perception.

## Introduction

Pitch is a fundamental auditory property that is used to analyse music, speech and auditory scenes. Rising and falling pitch contours in speech assist to establish prosody and improve speech intelligibility^1^. Peripheral neural correlates of pitch have been studied for over a century. However, it is still unclear what information from the auditory periphery is actually used by the auditory cortex to extract pitch. Scientific disputes revolve around the question of whether a pitch is coded by place cues in the basilar membrane, by temporal features of auditory neurons' spiking activity, or by a mix of the two^2,3^.

The significance of primary auditory neuron spike time cues in conveying pitch and speech information has attracted renewed interest^4^, in part because the lack of temporal coding in cochlear implants may explain some deficits experienced by cochlear implant users in perceiving music and pitch contours in speech^5–9^. Thus, in the present study we focus on "purely temporal" pitch perception, which we define as a pitch that can only be derived from the temporal response of primary auditory neurons.

Previous studies from our laboratory have investigated the peripheral neural code for perceived vibrotactile frequency. We have demonstrated, using psychophysical and electrophysiological techniques, that the most important temporal feature shaping perceived vibrotactile frequency or tactile pitch was the duration of the silent gap between two bursts of neural activity. We termed this interval the burst gap, and have shown that it is the dominant factor determining the perception of frequency, as opposed to either the class of afferent fibres activated, the mean spike rate or periodicity as thought previously^10–13^. This fits with the emerging narrative that the importance of temporal aspects of spiking activity appears as a common feature among sensory systems^14^. Given that temporal frequency channels in audition and touch have been demonstrated to be linked^15^, and certain tactile analysis mechanisms are thought to be analogous to those in the auditory system^16^, we now explore the auditory system to look for an equivalent neural coding strategy.

Previous studies have identified the importance of inter-spike intervals in conveying auditory pitch using temporally-structured acoustic pulse trains^17,18^. The autocorrelation theory^19^, and modern versions of it^20,21^ that take into account important aspects of peripheral processing including filtering, both assume that the auditory system analyses the intervals between each pulse and every other pulse (second order intervals) in acoustic pulse trains to extract pitch information. Kaernbach and Demany^22^, in contrast to autocorrelation models, claimed that the auditory system is only sensitive to first-order gaps between successive pulses, which is consistent with another study that indicated that pitch of a bandpass-filtered pulse train might simply be related to the mean pulse rate—as deleting random pulses from a pulse train lowered its pitch^23^. More recent findings agree that temporal pitch is derived from a weighted sum of the first-order intervals present in the stimulus train, with the greatest weight contributed by the longer inter-pulse interval^17,24^. Complex acoustic pulse trains, in particular periodic bursts of multiple pulses, however, are yet to be investigated to better comprehend the temporal neural correlates of pitch.

In this study, we sought to understand whether it is the overall pulse rate, periodicity, or any other time features within trains of pulses that determine perception of temporal pitch. Unlike previous pitch perception studies, we used complex 1 s acoustic pulse trains consisting of periodic bursts of multiple pulses. We probed the perceived pitch elicited by each train in psychophysical experiments involving normal-hearing human subjects. Stimuli that varied purely in temporal pitch were produced using acoustic trains of brief auditory pulses—each pulse being a 5 kHz (1 ms) Gaussian-modulated sinusoidal wave that should stimulate a fixed population of auditory fibres, thus ruling out cochlea place-based cues for pitch. Each brief auditory pulse should drive a sufficiently large population of cochlear neurons to respond in a synchronised manner^25^. We controlled the spiking pattern of 5 kHz responding cochlear neurons by temporally structuring these pulses in a train.

Understanding how the auditory system extracts pitch from temporal features of a pulse train could aid in the development of innovative cochlear implant signal-processing strategies. For example, fine-tuning in pitch perception could be achieved by varying temporal characteristics of electric pulses fed to an electrode stimulating a fixed locus in the cochlea.

## Materials and methods

The study was a controlled laboratory experiment involving behavioural measurements of the human ability to discriminate pitch of temporally structured acoustic pulse trains. The experimental protocols were approved by the Human Research Ethics Committee of the University of New South Wales, Australia (approval no. HC210031), and all experiments were performed in accordance with the guidelines and regulations of the Declaration of Helsinki.

### Subjects

Thirteen healthy volunteers (aged 18–40, 6 females) without any known history or presenting clinical signs of auditory disorders, screened via questionnaire, participated in the study. All participants provided written informed consent before conducting experiments. The sample size was determined by pilot studies to estimate effect size, and according to accepted practice in psychophysical experiments.

### Acoustic pulse train generation

Auditory pulse trains with desired temporal characteristics were generated using MATLAB (Mathworks, Natick, MA, USA) and Spike2 (Cambridge Electronic Design, Cambridge, UK). The stimulus waveforms were then converted to analogue voltage signals using a Power 1401 (CED, Cambridge, UK) and delivered by wired Bose QuietComfort 35 noise-cancelling headphones (Bose, USA).

Each acoustic pulse was a 1 ms, fixed amplitude, Gaussian-modulated 5 kHz sinewave, which would excite a fixed population of cochlear neurons. Custom Spike2 and MATLAB scripts controlled the delivery of pulsatile stimuli, and recorded the button presses of the subject. The timing of these action potentials in the activated neurons was manipulated by the temporal structuring of pulses in 1 s trains. Acoustic test pulse trains with characteristic temporal features are illustrated schematically in the respective experiment section along with the obtained psychophysical data.

### Psychophysical experiments to measure pitch

**Figure 1:**
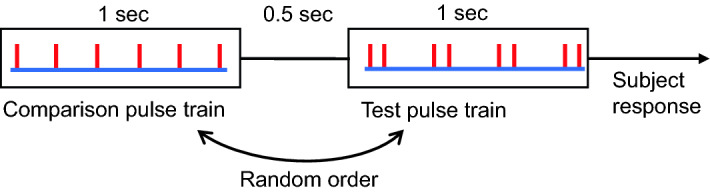
Two-interval force-choice method.

The loudness of individual pulses was optimised for each subject. For optimisation, a regular pulse train (40 Hz) was used. The pulse amplitude was increased in steps of 0.01 V, starting from 0.05 V, brief samples of the pulse train were delivered, and the procedure was repeated until the pulses were clearly heard and distinguishable but not uncomfortable for protracted listening. The determined stimulation amplitude was kept constant across all experiments for a subject. The perceived pitch of each test pulse train was determined using a two-interval forced-choice paradigm (Fig. 1) as in our previous tactile studies^11,13^. A test train was compared against six isochronous acoustic pulse trains (individual pulses evenly spaced) of different frequencies (pulse repetition rates). On each trial, the subject listened to a pair of stimuli, a test and one of the six comparison stimuli (isochronous pulse train), delivered for 1 s each, separated by 0.5 s, in random order. Subjects then had to indicate which stimulus had a higher pitch by pressing one of two buttons. Subjects were instructed to ignore any changes in the quality, loudness or intensity elicited by the pulse trains if such changes were to occur and to focus specifically on the pitch. Subjects’ responses, indicated by button presses, were acquired by the Power1401 and recorded in Spike2 for further analysis. Before actual data collection began, a brief practice session was conducted to familiarise subjects with the psychophysical task (twelve trials, both test and comparisons were regular trains).

To obtain psychometric curves, each test stimulus was compared twenty times against each of six different isochronous comparison frequencies, giving rise to 120 trials per test condition. The 120 trials were randomised within each test condition and between subjects. At each comparison frequency, the proportion of times the subject responded that it was higher in pitch than the test stimulus was determined (P_H_). Next, the logit transformation (ln(P_H_/(1 − P_H_))) was applied to the acquired data to produce a linear psychometric function^26^. The perceived pitch or apparent frequency was then taken as the point of subjective equality (PSE), the comparison frequency value that has an equal chance of being judged higher or lower than the test stimulus. It was determined as the frequency at the zero crossing of the logit axis from a regression line fitted to the logit transformed data.

### Statistical analysis

The R^2^ of the regression fits applied to the logit transformed psychophysics data was computed for each experiment. A one sample two-tailed t-test (n = 13) was used to test whether the experimentally obtained mean PSE value for each test stimulus in each experiment differed from its periodicity predicted and rate predicted value. A one-way repeated measures ANOVA compared PSEs across stimuli in each experiment. A two-way repeated measures ANOVA and post hoc Šídák's multiple comparisons was used to compare PSEs between experiment 1 and 2. Prism 8 (GraphPad Software, USA) was used for these analyses.

## Results

This study consisted of a series of three linked experiments. The goal was to see if the temporal structure of auditory pulse trains affects pitch perception, and if so, what temporal features within pulse trains determine the perceived pitch.

### Does the temporal structure of acoustic pulse trains affect the perception of pitch?

The first experiment tested whether the temporal structure of 1-s acoustic pulse trains affected the perception of frequency or pitch.

**Figure 2:**
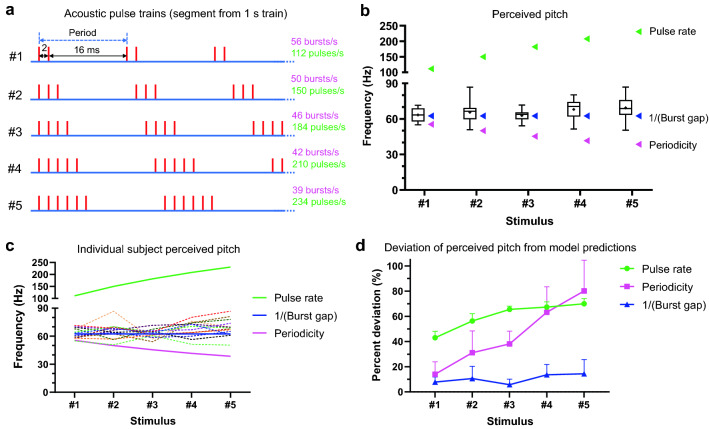
Schematic representation of stimulus patterns and the respective stimulus perceived frequency or pitch obtained in psychophysical tests. (**a**) Acoustic pulse trains with periodic bursts of 2–6 pulses, individual pulses spaced 2 ms apart. Each red vertical line indicates an auditory pulse. The stimuli had their own mean spike rate and burst rate, but the interval between successive bursts in every train was fixed at 16 ms. (**b**) Boxplots with whiskers represent the point of subjective equality (PSE) values obtained in psychophysical experiments for the corresponding stimulus illustrated in panel (**a**) (n = 13). The box extends from 25 to 75th percentiles; the whiskers extend to the minimum and maximum values. A dot within each box indicates a mean PSE (n = 13) value. Arrowheads indicate predicted PSE values for each pulse train if judgments were based on pulse rate (green arrowheads), periodicity/burst rate (pink arrowheads), and reciprocal of the inter-burst interval (1/Burst gap, blue arrowheads). (**c**) Individual subjects’ PSE values represented by dashed lines, solid lines indicate rate and periodicity predicted PSE values for each stimulus. (**d**) Deviation of subjects’ PSEs (n = 13) for each test stimulus from its rate, periodicity and burst gap predicted values, expressed as a percentage of the predicted value. Each data point represents mean and SD.

Five different 1 s auditory pulse trains consisting of periodic bursts of 2–6 pulses (Fig. 2a, stimuli 1–5) had their apparent frequency (or PSE) determined using a two-alternative forced-choice paradigm. The individual pulses within a burst were spaced 2 ms apart. Each test train had its own periodicity and pulse rate, but all the test trains had the same 16-ms interval between the end of one burst and the start of the next (inter-burst interval or burst gap). The isochronous comparison frequencies used to assess PSEs ranged from 30 to 100 Hz.

Were the pulse rate to determine the perceived frequency, there would be significant differences between the perceived frequencies for the five stimuli (ranging from 112 to 234 Hz; green arrowheads, Fig. 2b). Alternatively, if perceived frequency is shaped by a temporal component of the spike train related to its periodicity, such as the burst rate, perceived pitch would correspond to the individual train burst rate (ranging from 39 to 56 Hz; pink arrowheads, Fig. 2b). The apparent frequency of individual test trains was obtained after logit transformation of the respective psychometric data. The R^2^ of the regression fits was 0.93 ± 0.07 (mean ± SD). Individual subject apparent frequency is depicted as dashed lines in Fig. 2c. Neither the pulse rate nor the burst rate/periodicity could explain the experimentally observed apparent frequencies for the five test trains (boxplots, Fig. 2b). The experimentally obtained mean PSE value for each test train was significantly different from its periodicity predicted value (p = 0.0003 for stimulus #1, p < 0.0001 for the rest of the stimuli; one sample two-tailed t-test) and pulse rate predicted value (p < 0.0001 for each test stimulus).

Interestingly, the observed mean PSE values showed little difference across the test trains (F (2.771, 33.25) = 2.738, p = 0.063; RM one-way ANOVA), and the only stimulus parameter that closely matched the perceptual experience was the reciprocal of individual train inter-burst intervals which was fixed across stimuli (Fig. 2b, blue arrowheads, 62.5 Hz). The burst-gap model was observed to be the best predictor of perceived pitch among the three models (Fig. 2d). The discrepancy between burst gap predicted value and experimentally obtained mean PSEs ranged 0.3–6.8 Hz. Even the highest mismatched values (PSE 69.3 Hz vs 62.5 Hz burst-gap predicted for stimulus #5) are close to limit of pitch discrimination as expected from the Weber fraction which has been reported as 2–5.5% for regular click rates ranging 50–200 Hz^27–29^.

The data provide evidence that the inter-burst interval (burst gap), rather than pulse rate or periodicity, was the most salient time element in the auditory pulse trains that shaped pitch. The inter-burst interval relates to the silent or quiescent phase between bursts of auditory neural activity.

### Does pulse count within burst influence the perceived pitch?

Even though the participants indicated that pitch perception was clear and that they could make a judgement regardless of other cues, we had to rule out the possibility that the variation in the number of pulses within bursts served as an intensity cue, confounding pitch perception. A second experiment was designed to determine whether the pulse count within the bursts biased subjects’ frequency judgements.

**Figure 3:**
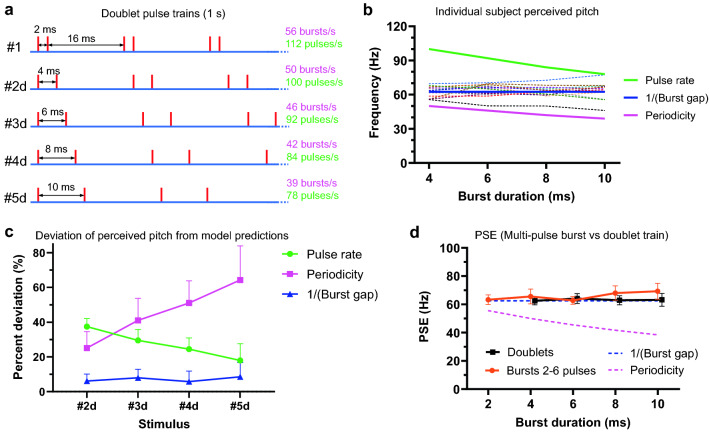
Pulse count within bursts of trains marginally influences the perceived pitch. (**a**) Schematic representation of acoustic pulse trains used in experiment 2, each vertical line indicates the timing of a pulse. The index ‘d’ stands for doublet train. It distinguishes corresponding stimuli from those in experiment 1. Trains are identical in burst duration, and inter-burst interval (fixed across the stimuli). (**b**) PSEs elicited by the stimuli illustrated in panel (**a**). Dashed lines represent experimentally obtained individual subjects’ PSEs. Solid lines indicate predicted PSE values by pulse rate (green), reciprocal of burst gap (blue), and periodicity/burst rate (pink). (**c**) Deviation of subjects’ PSEs (n = 13) for each test stimulus from its rate, periodicity and burst gap predicted values, expressed as a percentage of the predicted value. Each data point represents mean and SD. (**d**) Comparing PSEs evoked by corresponding stimuli in experiments 1 and 2. PSEs for perceived pitch of doublet trains illustrated in panel (**a**) (black line with squares, n = 13). PSEs for stimuli in experiment 1 (illustrated in Fig. 2a) are plotted for comparison (orange line with circles, slightly shifted horizontally, n = 13). Dashed lines represent PSE values predicted by the burst gap model (blue) and periodicity (pink). Error bars denote ± 95% confidence intervals.

The stimuli tested in the second experiment are illustrated in Fig. 3a; they differ from experiment one by having doublets (2 pulses per burst) instead of multi-pulse bursts. The burst duration of a given stimulus (#2d–#4d, ‘d’ referring to doublet train) was identical to that of the matching multi-pulse burst stimulus (#2–#4) in experiment 1. The inter-burst interval was fixed at 16 ms, as in experiment 1. The same psychophysical method was used to determine the perceived pitch elicited by each doublet train in thirteen subjects (Fig. 3b; dashed lines represent individual subjects). The R^2^ of the regression fits on experimentally obtained logit transformed psychophysical data was 0.94 ± 0.06 (mean ± SD).

The predicted PSEs from pulse rate and periodicity models are also plotted for comparison. The experimentally observed mean PSE value for each test stimulus was significantly different from its periodicity predicted value (p < 0.0001 for each test stimulus; one-sample two-tailed t-test) and pulse rate predicted value (p < 0.0001 for each test stimulus). As in experiment 1, observed PSEs showed little difference across test trains (F (1.981, 23.77) = 0.4255, p = 0.65; RM one-way ANOVA). The better predictor of the perceived pitch than rate or period was the reciprocal of the inter-burst interval in stimulus trains (burst-gap model) (Fig. 3c).

When comparing perceived pitch of this set of stimuli to corresponding stimuli with multiple pulses in experiment one (Fig. 3d), the two stimulus types produced very similar results. The pulse count within a burst accounts for only 5% of total variation (F (1, 12) = 6.058, p = 0.03; two-way RM ANOVA excluding stimulus #1), while burst duration accounts for 2.35% (F (3, 36) = 1.32, p = 0.28), and interaction (pulse count x burst duration) for 3.9% (F (3, 36) = 2.749, p = 0.06). Post hoc Šídák's multiple comparisons test showed significant difference only between stimulus #5 and #5d (adjusted p = 0.0158). Though there is a substantial variation in pulse rate, the difference in mean PSEs between stimulus #5d (78 pulses/s) and stimulus #5 (234 pulses/s) is only 6.08 Hz (95% CI 0.9–11.27). This suggests that under these conditions, the pulse number within bursts up to 10 ms duration only has a marginal effect on the perceived pitch. Instead, pitch closely corresponds only to the quiescent period between bursts, and was found not to be the function of the rate or periodicity of stimulus pulses.

### Does the burst gap code prediction hold for a shorter inter-burst interval?

**Figure 4:**
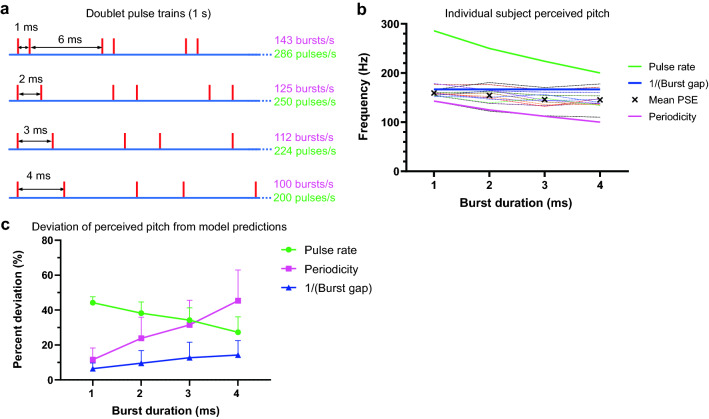
Pitch perception at 6 ms inter-burst interval. (**a**) Schematic representation of doublet trains with varying burst duration but consistent inter-burst interval (6 ms). (**b**) PSE values obtained in psychophysical tests. Dashed lines represent individual subjects’ PSEs (n = 13), solid lines indicate predicted PSEs if pitch judgements were based on pulse rate (green), the inverse of the inter-burst interval/burst gap (blue), and periodicity (pink). (**c**) Deviation of subjects’ PSEs (n = 13) for each test stimulus from its rate, periodicity and burst gap predicted values, expressed as a percentage of the predicted value. Each data point represents mean and SD.

We were curious to test if the inter-burst interval, which we discovered to be the most critical temporal characteristic that determined pitch, was still true at a shorter interval. The inter-burst interval was set at 6 ms across all pulse trains, and burst duration was varied from 1 to 4 ms. Stimuli had their own pulse rate and periodicity (Fig. 4a). The isochronous comparison frequencies used to assess PSEs ranged from 95 to 200 Hz.

The same psychophysical method was used to determine perceived pitch elicited by the doublet trains. The mean R^2^ of the regression fits applied to the psychophysical data was 0.92 (± 0.07, SD). Individual subjects’ perceived pitch values for the stimuli are represented by the dashed lines in Fig. 4b, plotted against the stimulus burst duration. Solid lines represent predicted perceived pitch by pulse rate, burst gap and periodicity models. Twelve of the thirteen subjects closely followed the prediction from the inter-burst interval, although one appeared to follow the prediction from periodicity. The observed mean PSE for each test stimulus is significantly different from its periodicity predicted value (p < 0.001 for each test stimulus; one sample two-tailed t-test) and pulse rate predicted value (p < 0.001 for each test stimulus).

The mean perceived pitch (n = 13) of four stimuli corresponded to that of isochronous pulse trains having inter-pulse intervals of 6.3 (95% CI 6.0–6.5), 6.5 (6.1–6.9), 6.8 (6.4–7.3), and 6.8 (6.4–7.4) ms (1–4 ms burst stimuli respectively). This was a close match to the inter-burst interval, which was fixed at 6 ms, as opposed to respective stimulus complete period (burst duration + inter-burst interval) or the mean of two intervals. The biggest deviation of actual PSE from burst gap predicted value (166.7 Hz) was observed for stimuli with more extended burst envelops—3 ms (mean 146.1 Hz, 95% CI 136.6–155.6) and 4 ms (145.4 Hz, 95% CI 134.8–156), both around 12.5% lower than predicted (Fig. 4b). The mean PSEs were different across the four stimuli (F (2.159, 25.91) = 9.626, p = 0.0006; RM one-way ANOVA) unlike in experiments 1 and 2, indicating the effect of burst duration. Still, the results are most consistent with an explanation of perceived pitch derived from the inter-burst interval rather than rate or period (Fig. 4c).

## Discussion

This study used brief 5 kHz pulses to excite a fixed set of cochlea afferents, which eliminated place-of-excitation as a cue for pitch. The perceptual pitch evoked by pulse trains containing bursts of various temporal structures was examined to determine the key time feature that determines the perceived pitch. Burst firing—the intermittent firing of high-frequency action potentials—is a prominent feature of various sensory neurons^30^. Bursts are thought to play a vital role in the reliable transmission of neuronal information as they can elicit long-term synaptic plasticity and encode more information than single isolated spikes^31^. Furthermore, bursts provide an extra dimension to the neural codes: the literature suggests that bursts and spikes within bursts can form a parallel code—in which they code for different stimulus features in the same spike train^32^.

### Duration of silent period between successive bursts of neural activity encodes the temporal pitch: an analogy with touch

The present study demonstrates that when a fixed population of peripheral auditory neurons were stimulated in periodic bursts, the perceived pitch best corresponded to the silent interval between successive bursts, which we call the burst gap, rather than to the complete period (burst duration + burst gap) or the average of the inter-pulse intervals present. Bursts with durations up to 10 ms were perceptually resolved as single auditory events, with spikes hidden within bursts minimally influencing the perceived pitch (Figs. 2b and 3d). Burst gap coding was shown to operate for perceived frequencies up to 165 Hz, where burst durations between 1 and 4 ms had minimal influence on the perceived frequency. At a shorter burst gap (6 ms) increasing the burst duration may begin to influence frequency perception, as had been previously observed in the tactile system^10,33^.

These findings are consistent with what we have previously reported in relation to the perception of vibrotactile pitch. Primary tactile afferents discharging periodic bursts of multiple spikes (resembling responses to high-amplitude vibration) encoded stimulus frequency in the silent period between successive bursts^10,11^. When multiple spikes were grouped into a “burst” of a maximum duration of 15 ms, the number of spikes within each burst did not affect frequency perception^13^. Indeed, the number of spikes within a burst could potentially correlate with an additional stimulus feature, such as the stimulus intensity. Relating this to the natural stimulation of the auditory system, the rising phase of each sound wave cycle could elicit bursts of spikes in a bundle of the most sensitive auditory fibres, with the number of spikes per burst determined by the amplitude and the timing between bursts contributing pitch information that may supplement the place code. The burst-gap code appears to be a shared feature for pitch analysis across audition and touch. The emerging literature supporting this notion has demonstrated anatomical connectivity^34^ and frequency perceptual interactions^15,35^ between auditory and somatosensory systems, suggestive of a neural and functional link. For example, significant ipsilateral connections between somatosensory (primary and secondary) and primary auditory cortices were shown in humans^34^ and non-human primates^36^. Functionally, auditory cues exerted biases on the perception of low and high-frequency tactile vibrations^15,35^, and reciprocally, tactile cues biased auditory frequency perception^37^.

Codes based on spike timing have previously been shown to transmit more information than mean rate codes^38^. The precise spike times in peripheral auditory neurons were found to contain the information required to account for human discrimination of minor frequency changes^39–42^. Sound intensity (subjective loudness) was more correlated to temporally coarse spike-rate information in auditory peripheral neurons^43–45^, similar to the encoding of tactile stimulus intensity^46^. Time-based pitch coding may be recoded at higher levels of the nervous system as temporal fidelity degrades across successive synapses which makes spike timing a less viable code at a cortical level^44^.

In analysing the relation between perceived pitch and auditory nerve impulse pattern, the distinction between periodicity and pulse intervals has been the subject of enquiry for some time. Periodicity was shown not to be uniquely related to pitch^18,39^. Whitfield^18^, in his experiment, assessed the pitch evoked by a pulse train with alternate intervals of 4.7 and 5.3 ms. Auditory single nerve fibres recordings were made in anaesthetised guinea pigs, and it was verified that predominant inter-spike intervals matched the pulse intervals. Subjective listening tests revealed that human observers did not hear pitches corresponding to these intervals (213 and 189 Hz) but instead heard pitches around 200 Hz (corresponding to a 5 ms interval). This indicated that time intervals between successive nerve impulses were not necessarily a direct correlate of pitch. More recent studies^17,47^ in normal and cochlear implant users, showed that when no place-excitation cues were available to the subjects, acoustic and electric pulse trains with alternating 4 and 6 ms intervals evoked a pitch percept equivalent to a 5.7 ms interval. The observed pitch was longer than the mean interval (5 ms) and shorter than the 10 ms total period. These results were not consistent with predictions from the mean rate model, or the autocorrelogram model that operates on higher-order intervals. When we tested a 4–6 pulse train (bottom stimulus in Fig. 3A), perceived pitch corresponded to 6.9 ms—which agrees with these findings in being longer than mean interval and shorter than a total period. The possible reason for the discrepancy (5.7 ms vs 6.9 ms) may stem from the methodological differences: Carlyon’s group used 400 ms bandpass filtered acoustic trains that were attenuated and mixed with pink noise before being delivering to normal hearing listeners. The shorter duration of stimuli^48^ and the background of continuous pink noise^49^ in their study may have influenced the discriminative tasks.

### Importance of peripheral spike timing cues

It is argued that our ability to discern between two different pitches is far finer than what the fundamental place theory of pitch would resolve. We can discern two tones differently under ideal settings if their repetition rates differ by just 0.2 percent (one thirtieth of a semitone)^41^. The sharpness of tuning (the range of frequencies to which each place responds) of each place on the basilar membrane, on the other hand, is around 15% of the tuned frequency^50,51^. As a result, the membrane's tuning may not be fine enough to discern between frequencies that are so close together. Therefore, the most common model for the sensitivity for the fine discrimination in pitch perception is that it may rely on the temporal structure of spikes in activated fibres^52,53^, although it should be noted that some authors have offered alternate interpretations^54^.

Animal research based on frequency analysis in the cochlea has revealed that the place code changes systematically as a function of pure tone sound amplitude^55–57^ as well as pitch, indicating that it lacks the resilience required to fully explain pitch perception (in humans), which is nearly independent of sound intensity. Furthermore, impairment of spectral analysis in the cochlea in some individuals was not correlated with deficits in speech discrimination^58^.

Auditory nerve injuries, in particular demyelination, cause an increase in neural conduction time^59^, as indicated by prolonged compound action potential duration recorded directly from the exposed nerve after surgical manipulation of the eighth cranial nerve^58^. Temporal dispersion of neural activity among active fibres would almost certainly negatively impact the ability of higher auditory centres to use spike timing cues for pitch discrimination. It is known that auditory nerve injury produced by acoustic tumours^60^ and surgical manipulations^61^ impedes speech discrimination more than a similar hearing loss caused by cochlear injuries^58^, which suggest the importance of temporal coherence in auditory fibre activity.

### Auditory neurons tuned to a high frequency can also convey low-frequency pitch

Apart from the fact that the temporal spiking feature of cochlear neurons shapes pitch, our results also revealed a remarkable finding that cochlear neurons tuned to high-frequency sound waves (5 kHz in this case) could effectively convey the pitch of low-frequency pulse trains. We observed a similar phenomenon in the tactile system relating to the perceived frequency of mechanical pulsatile stimuli. We showed that tactile afferents tuned to high sinusoidal frequencies (100–800 Hz, Pacinian fibres) could readily elicit vibratory percepts of mechanical pulse trains of much lower frequency (20–40 Hz). Importantly, the vibratory percept evoked was analogous to that elicited by low frequency preferring non-Pacinian fibres, which shows that spiking pattern of active afferents, rather than afferent type, shapes the perceived frequency^62^. Interestingly, the auditory data presented here suggest that peripheral inputs from areas of the basilar membrane other than the resonant area may also contribute to pure tone pitch perception, as neurons that were tuned at one frequency could also convey other frequencies. This accords with the natural high amplitude stimulation, for instance, as loudness of a tone increases—the mechanical tuning curve of the basilar membrane grows wider^63,64^, that leads to the progressive recruitment of afferents of varied optimal frequencies and afferents sensitive to the centre frequency saturate^65^. The auditory cortex may then deploy a rate-based cortical population coding scheme to extract frequency or pitch^44^.

### Implications for cochlear implants

Pitch information delivered by implanted electrodes employing differential stimulation of auditory nerve fibres appears to be limited^66^. Therefore, for precise pitch discrimination, cochlear implants could also rely on the temporal patterning of electrical pulses in stimulating electrodes^67,68^. In studies of both haptic displays^69^ and neural prostheses^70^, burst stimulation has been progressively employed as a strategy for transmitting sensory information.

Mimicking natural complex spectrum analysis in the cochlea by increasing the number and selectivity of electrodes in implants is not achievable in the imminent future^71^ despite innovative approaches to improve electrical access^72^, due to spatial limitations that restrict the specificity of the population of afferents activated. As an alternative, reproducing diverse temporal firing patterns in activated auditory neurones to trigger pitch gradations would be reasonably straightforward with current technology. In some initial investigations of pitch perception in cochlear implant users, the temporal cues delivered to the individuals were manipulated. For example, in studies where melodies were delivered to a single electrode (no place cues), subjects were able to detect and differentiate melodies^73,74^. Similarly, the fact that coding of vowel waveforms in the discharge pattern of single auditory nerve fibres^75^ is more robust than spectral coding^65^ backs up the idea of using temporal cues in implants. Both suggest the success of cochlear implants for satisfactory pitch discrimination could be achieved without requiring precise differential stimulation of auditory afferents.

## Conclusion

The temporal structure of acoustic pulse trains influences the perception of pitch. When acoustic pulses are structured into periodic bursts of multiple pulses, perceived pitch is best explained by the interval between successive bursts, as opposed to the pulse rate or burst rate (periodicity). The burst stimulation method described here could be employed in cochlear implants to deliver pitch information in parallel with other sound features encoded by intra-burst pulse characteristics.

## Data Availability

The datasets generated and/or analysed during the current study are available from the corresponding author on reasonable request.
